# Molecular Compatibility and Hydrogen Bonding Mechanism of PES/PEI Blends

**DOI:** 10.3390/polym14153046

**Published:** 2022-07-27

**Authors:** Yuanlu Zhu, Weixing Wu, Ming Gao, Jiangyi Yan, Beifu Wang

**Affiliations:** 1 School of Naval Architecture and Shipping, Zhejiang Ocean University, Zhoushan 316000, China; xiaoluer113@163.com (Y.Z.); gaoming97@163.com (M.G.); 2 School of Petrochemical Engineering and Environment, Zhejiang Ocean University, Zhoushan 316000, China; wwxyixingfeng@163.com (W.W.); jiang1842022@163.com (J.Y.)

**Keywords:** polyethersulfone, polyetherimide, blend, hydrogen bonding, compatibility

## Abstract

The development of high-performance polymer membranes has sparked a lot of attention in recent years. Polymer blending is a potential method of modification. A limitation, however, is the compatibility of blends at the molecular level. In this investigation, polyethersulfone/polyetherimide hollow fiber membranes were prepared by the solution blending method. Compatibility, hydrogen bonding, crystallinity, microstructure, hydrophilicity, mechanical properties, and transmissibility of blended membranes were also characterized. The compatibility and hydrogen bonding action of the two components were confirmed by DSC, FTIR, XPS, and XRD. The structure exhibits a C−H···O interaction motif with the sulfone group acting as a hydrogen bond acceptor from a methyl C−H donor. The π–π stacking between the two polymers arranged molecules more orderly, resulting in enhanced intermolecular interactions. Compared to polyethersulfone hollow fiber membranes, the hydrophilic, mechanical properties, and rejection rate of the blended membranes are more effectively enhanced. Self-assembly of the host polymer with a polymer capable of forming hydrogen bonds to construct controllable blends is a crucial and proven method.

## 1. Introduction

With the higher requirements of membrane materials and membrane properties in the field of membrane technology, conventional membrane materials have been unable to meet the needs of technological development. Blending membranes supplement the categories of existing membrane materials; integrate the properties of a variety of membrane materials; overcome the shortcomings of single-membrane materials; create membrane materials with different characteristics with synergistic and complementary advantages; and provide more diversified choices in practical applications [1]. The proportion of the polymer blend has been verified to affect the hydrophilicity, pore structure, and mechanical properties of the membrane. A limitation, however, is the compatibility of blends at the molecular level [2,3,4]. Special interactions between polymer molecules, such as intermolecular hydrogen bonds, ion–ion, dipole–dipole, charge-transfer complexes, and acid–base interactions, have been used by many researchers to improve mix compatibility [5,6,7]. The strongest intermolecular interaction is the hydrogen bond, and the formation of an intermolecular hydrogen bond can restrict the degree of freedom of the original-component molecular chain and control the aggregation structure of the polymer at the molecular level [8]. The application of hydrogen bond self-assembly of controlled polymer materials is possible. Han et al. [9] investigated the molecular interaction between TPU and PPC, finding that the two components formed a hydrogen bond, which strengthened the blend’s compatibility and mechanical qualities. Sarhan et al. [10] used Cs and PEG in the correct proportions. The hydroxyl group of PEG is an electron donor, which means it can generate hydrogen bond interactions with Cs to promote compatibility.

The PES molecular structure (Table 1) contains the sulfone group, which has good oxidation resistance and thermal stability, and the ether bond enhances the flexibility of the molecular chain. Sulfone groups, ether bonds, and benzene rings produce a conjugated system with chemical characteristics that are extremely stable [11]. It can be used for a long time at a high temperature of 150–170 °C and has a spinning ability suitable for hollow fiber form [12]. The compatibility of polyethersulfone (PES) blends with other polymers has been proven [13,14,15]. Cha et al. have reported that PES and polyimide (PI) have good blending compatibility, but PI is difficult to mold and expensive. Polyetherimide (PEI) is a kind of polymer formed by introducing ether bonds into the polyimide chain, which overcomes the shortcomings of PI and possesses both the rigid structure of aromatic amide groups and the processability provided by ether bonds [16]. The molecular structure is shown in Table 1.

There are few studies on PES/PEI blends. By the phase inversion method, Govardhan et al. [17] prepared the hollow fiber membrane composed of polyethersulfone/polyetherimide blends. The membranes have uniform morphology, hydrophilicity, and stability. The compatibility of the blends was confirmed by experiments and tested in a laboratory membrane unit for surface water reclamation. Zhao et al. [18] also used the phase inversion method to prepare PES/PEI blend hollow fiber membranes. Pervaporation (PV) was used to investigate the removal of volatile organic compounds (VOCs) from water solutions through PES/PEI blend hollow fiber membranes. The blended membranes showed high VOC/water selectivity and permeation flux to the VOC aqueous solutions. The compatibility and intermolecular hydrogen bonding of PES/PEI blend hollow fiber membranes were discussed by combining the test analysis and theoretical calculation in this paper.

## 2. Experimental

### 2.1. Materials

Polyethersulfone (PES, A.R) was provided by German manufacturer BASF Co., Ltd., Ludwigshafen, Germany; polyetherimide (PEI, A.R) was provided by American manufacturer SABIC Co., Ltd., Riyadh, Saudi Arabia; *N*,*N*-dimethylacetamide (C_4_H_9_NO, DMAc, A.R) was purchased from National Pharmaceutical Chemicals Co., Ltd., Beijing, China; and polyvinylpyrrolidone (PVP, A.R) was purchased from Yatai United Chemical Technology Co., Ltd., Suzhou, China. The molecular weight of the polymers used in the experiment is shown in Table 2.

### 2.2. Preparation of Blended Hollow Fiber Membranes

The hollow fiber membranes were obtained by solution blending. The medications for the experiment must be dried at 60 °C in a constant-temperature drying oven. PEI particles and *N*,*N*-dimethyl acetamide (DMAc) were carefully weighed and added to the reaction kettle at 80 °C, stirring continuously until PEI dissolved completely. The temperature was then reduced to 60 °C, and the polyethersulfone (PES) and polyvinylpyrrolidone (PVP) were introduced to the reaction kettle and mechanically agitated for 12 h to thoroughly combine them. The homogenized liquid was kept at room temperature for 12 h to defoam. Table 3 lists the particular experimental ratios. PVP as an additive was introduced into the solution to modulate the viscosity of the mixture when required.

**Figure 1:**
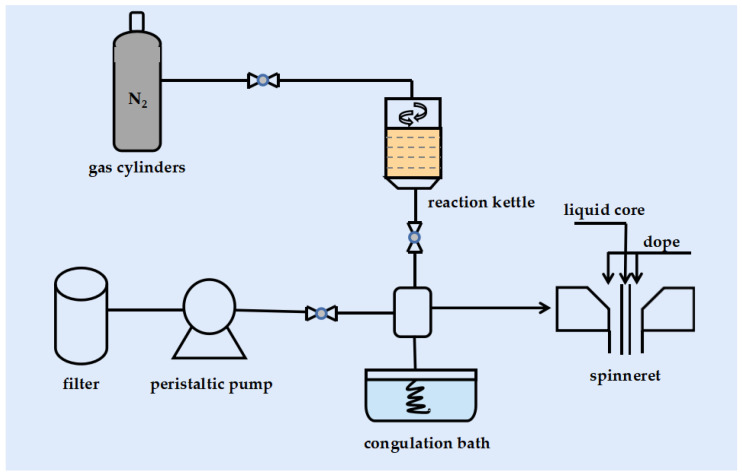
Flow chart of hollow fiber membrane spinning.

A vacuum pump was used to defoam the casting fluid again before spinning to guarantee that all bubbles were gone. The entire spinning process takes place at a temperature of 20–25 °C with a humidity of 60–65%. The casting solution and core were hydraulically fed into the spinneret at a pressure of 1.2 MPa. The core liquid consists of deionized water and the gel tank consists of water. The extruded new fibers enter the gel tank and solidify to form hollow fiber membranes. The distance between the spinneret and the gel tank was 3 cm, the inner and outer diameters of the spinneret were 0.72 mm and 2.0 mm, respectively, and the core liquid rate was 0.8 L/min. Care was taken to ensure that the hollow fiber was not tangled during the spinning process and that the pressure and internal water levels were constant to avoid air entrainment and fiber breakage. The spun hollow fiber membranes were immersed in deionized water for 48 h to remove residual solvents and dried for subsequent characterization. Figure 1 shows the spinning process of the hollow fiber membrane.

### 2.3. Membrane Characterizations

#### 2.3.1. DSC–TG

The glass transition temperature of PES/PEI membranes was studied using a differential scanning calorimeter (DSC 200-F3, NETZSCH, Selb, Germany) in a nitrogen atmosphere. About 8 mg of the dry membrane was sealed in an aluminum pan. It was kept at 20 °C for 3 min, then heated to 400 °C at a heating rate of 10 °C/min and maintained for 3 min to remove the thermal history, and then chilled to 20 °C (at a rate of 10 °C/min).

The PerkinElmer STA-8000 System analyzer was used to evaluate the thermal decomposition of various PES/PEI membranes by performing thermogravimetric analysis by heating samples of around 8.0 mg from 30 to 800 °C at a rate of 10 °C/min.

#### 2.3.2. FTIR

Fourier transform infrared spectroscopy-attenuated total reflectance (FTIR-ATR) measurement was carried out using a Thermo Scientific Nicolet iS20 Fourier transform infrared spectrometer. The membranes were cut along the longitudinal length of the fiber with a sharp blade, and a section of membrane with the inner surface of 30 mm × 50 mm was completely exposed as a sample. The samples were placed on the sample holder, and all spectra were recorded from 4000 cm^−1^ to 600 cm^−1^.

#### 2.3.3. XPS

X-ray photoelectron spectroscopy (XPS Thermos Scientific K-Alpha, Waltham, MA, USA) was used to investigate the surface chemical compositions of membranes. The range of survey spectra is from 0 to 1300 eV, and the C1s’ peak of high-resolution spectra was detected. XPS full-scan spectra were recorded within the range from 0 to 1300 eV with a pass energy of 150 eV with a monochromatic Al Kα X-ray source at 1486.6 eV.

#### 2.3.4. XRD

The crystal structure of PES/PEI membranes in the prepared membranes was determined by means of a wide-angle X-ray diffractometer (WAXD, DX-2700, Shanghai, China). The scanning parameters included the source intensity (40 kV/30 mA), λ (1.54 Å, Cu Kα line), source slit width (0.6 mm), increment rate (2°/min), and scanning range (5–40°).

#### 2.3.5. SEM

The morphology of the cross section was observed by a scanning electron microscope (SEM Gemini 300, ZEISS, Jena, Germany) with an accelerating voltage of 10 kV and 40 kV. The cross-sectional morphologies of various membranes were examined using scanning electron microscopy (SEM Gemini 300, ZEISS, Jena, Germany) with an accelerating voltage of 10 kV and 40 kV. When characterizing cross-sectional morphologies, the samples were prepared by fracturing membranes after fully cooling in liquid nitrogen. All samples were treated with Au/Pd sputtering and carefully handled to avoid contamination.

#### 2.3.6. Contact Angle Measurements of the Membranes

The hydrophilicity of the membrane was observed based on the water contact angle measurement (WCA). The WCA of the membrane was measured using an optical contact angle tester (OCA15Pro, Data Physics Instruments GmbH, Filderstadt, Germany) according to the sessile-drop method. Samples with a length of 100 mm were taken from each group, and deionized water (10 mL) was dropped evenly and slowly on the inner surface of the fiber through the instrument. The average was made by measuring parallel three times for each sample.

#### 2.3.7. Mechanical Property of the Membranes

The microcomputer-controlled electronic universal testing machine (CMT8501, Shenzhen, China) was employed to determine the breaking strength and breaking elongation of the membrane by measuring the stress–strain curve. Each group takes a specimen with a length of 100 mm, adjusts the upper and lower spacing of the jig to 25 mm, fixes both ends of the membrane, and stretches at a rate of 100 mm/min. Each sample was tested three times to obtain average strength.

#### 2.3.8. The Transportability, Anti-Fouling Performance of the Membranes

Pure water flux (PWF) was measured with an ultrafilter at 0.12 MPa pressure. The flux was equilibrated for the passage of the first 30 min of permeation, whilst the following 10 min of permeation was collected. Pure water flux was evaluated by the following:

(1) $$J=\frac{V}{A\cdot \Delta t}$$

where J is the pure water flux (L/(m^2^·h)), V is the volume of penetrated water (L), A is the effective area of the membrane (m^2^), and Δt is the recorded time (h). All experiments were conducted thrice to obtain the results presented in this study, which were an average value.

UV/visible spectroscopy (TU-1901, General analysis) was employed to measure the concentration of feed solution and permeation of the BSA solution at a wavelength of 280 nm. The feed solution was 0.1 g/L BSA solution. Then, the filtered solution was obtained in the same way as for water flux testing. The membrane rejection (*R*) was obtained by the following equation:

(2) $$R=\frac{C_{F}-C_{P}}{C_{F}}\times 100\%$$

where R is the percentage retention rate or BSA rejection, $C_{F}$ is the concentration of feed solution, and $C_{P}$ is the concentration of permeation.

## 3. Results and Discussion

### 3.1. Thermal Stability of the Membranes

**Figure 2:**
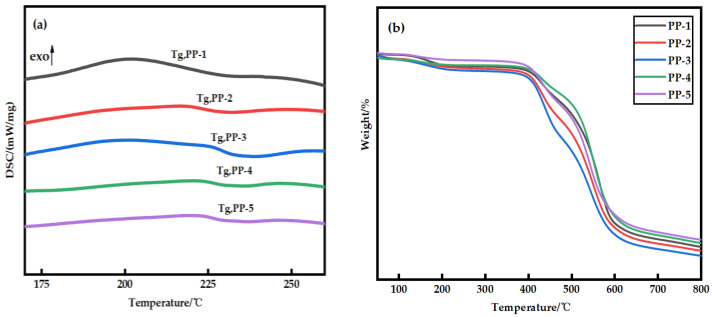
(**a**) Glass transition temperature of PES/PEI membranes with different ratios; (**b**) Thermogravimetric curves of PES/PEI membranes with different ratios.

**Figure 3:**
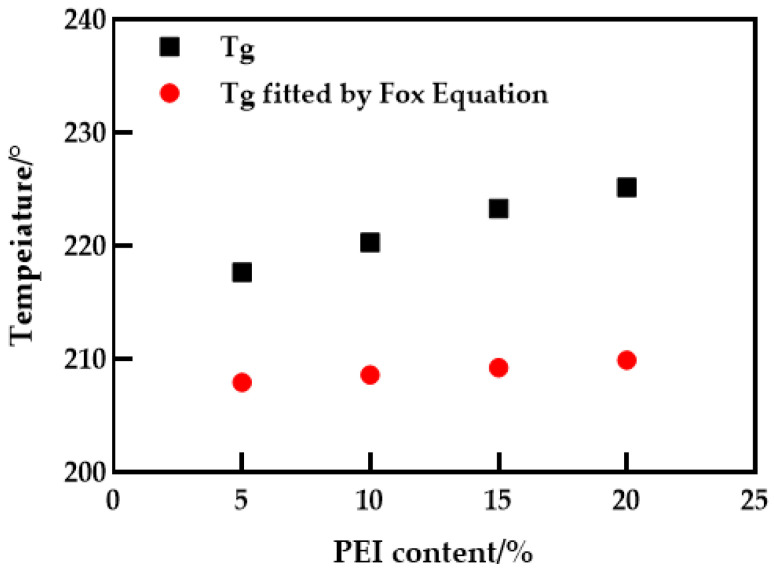
Theoretical and measured values of the glass transition temperature of the blending system.

The thermal stability of the blended membranes was studied using DSC–TG simultaneous thermal analysis, as shown in Figure 2. It can be seen from Figure 2a that the glass transition temperature (*T_g_*) of PES membrane is about 202.3 °C, and when PEI is added, *T_g_* increases to about 225.2 °C. It is noteworthy that the appearance of a single glass transition temperature associated with the composition indicates that only one phase transition occurs in the blend membrane. The change of compatible blend *T_g_* can be explained by the Fox empirical equation [19] (Equation (3)), as shown in Figure 3. Obviously, the Fox equation has the same general trend as the measured results. An interesting phenomenon is that the *T_g_* of the measured blend membrane is slightly higher, which may be due to hydrogen bonding. The subsequent FTIR also confirmed this conjecture. At the same time, the interaction between PES and PEI molecular chains also leads to a decrease in the activity of chain segments, which is consistent with the conclusion obtained in the literature [20]. According to Figure 2b, the decomposition temperature of the PES membrane and PES/PEI blend membranes showed a similar change trend, but the decomposition temperature increased slightly. Because the energy required to break a hydrogen bond increases as the number of hydrogen bond action points increases, the blend membrane’s thermal stability grows.

(3) $$\frac{1}{T_{g}}=\frac{W_{1}}{T_{g1}}+\frac{W_{2}}{T_{g2}}$$

where $T_{g}$ represents the glass transition temperature (K) of the blend, $W_{1}$ and $W_{2}$ are the mass percentages (%) of components 1 and 2, respectively, and $T_{g1}$ and $T_{g2}$ are the glass transition temperatures (K) of components 1 and 2, respectively.

### 3.2. Functional Group Composition of the Membranes

**Figure 4:**
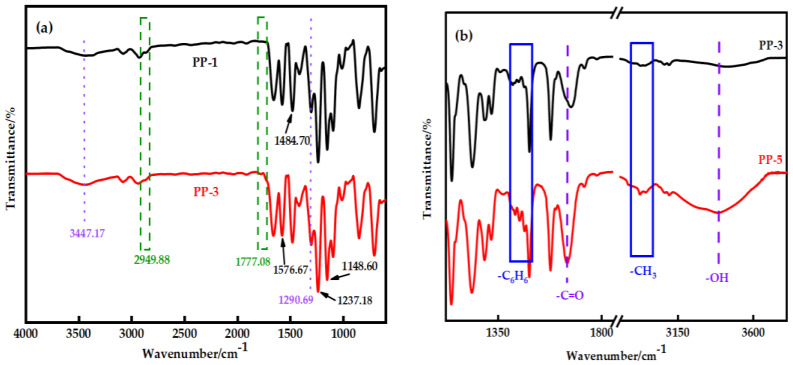

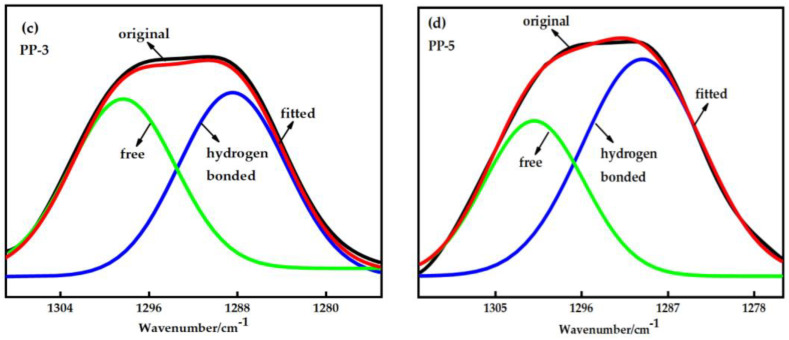
(**a**) Fourier infrared spectra of the PP-1 and PP-3 membranes; (**b**) The characteristic peaks of methyl and benzene rings are in PP-3 and PP-5 membranes; (**c**) Curve fitting results in the sulfone group stretching region of the PP-3 membrane; (**d**) Curve fitting results in the sulfone group stretching region of the PP-5 membrane.

Figure 4a shows the FTIR spectra of the membrane before and after modification. PES membranes show peaks at 1148.6 cm^−1^ and 1295.76 cm^−1^, corresponding to the symmetric and asymmetric tensile vibration characteristics of the sulfone group (-SO_2_^−^). The symmetric tensile vibration of the aromatic ether (-C-O-C-) bond appears at 1237.18 cm^−1^ as an evident peak. The absorption peaks at 1484.7 cm^−1^ and 1576.67 cm^−1^ belong to the skeleton vibration of the benzene ring group. Other wavelengths detected at 718.19 cm^−1^ and 797.17 cm^−1^ are phenyl groups [21].

**Figure 5:**
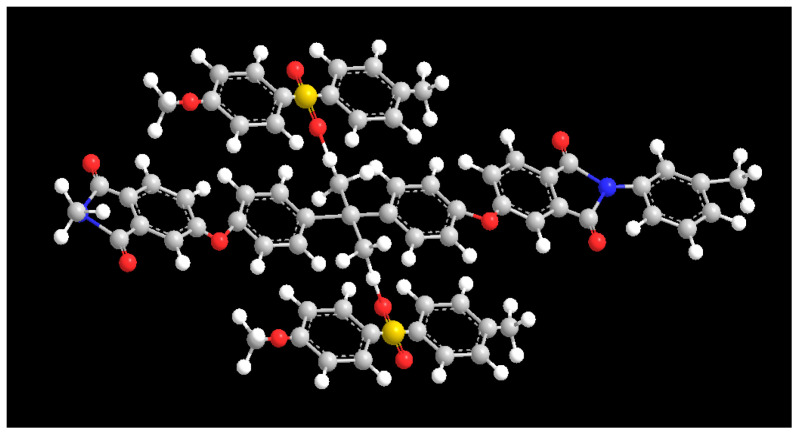
π–π stacking and hydrogen bonding model between PES and PEI.

It is worth noting that the asymmetric tensile vibrations of imide cyclocarbonyl (C=O) and methyl (-CH_3_) of PEI were represented by new absorption peaks in the PP-3 blend membrane at wavenumbers of 1777.08 cm^−1^ and 2949.88 cm^−1^, respectively. The appearance of new peaks in the blend membrane indicated that PES and PEI were effectively blended. Interestingly, the methyl group’s characteristic peak in PP-5 film moved to 2940.91 cm^−1^, and the benzene ring’s characteristic peak moved to 1473.94 cm^−1^, and the strength increased (Figure 4b). The widening and shifting of the absorption band in FTIR spectra confirmed the existence of an intermolecular interaction between PES and PEI. The structure (Figure 5) exhibits a C−H···O interaction motif with the sulfone group acting as a hydrogen bond acceptor from a C−H donor.

The formation of hydrogen bonds and the relative content of hydrogen-bonded sulfone groups can be characterized by measuring the infrared absorption peaks of hydrogen bond acceptors (sulfone groups) in the blends; that is, the number of hydrogen bonds formed between the two components of the blends can be obtained [22,23,24]. Figure 4c,d shows that the stretching frequency of the sulfone group is divided into two bands. These two bands can be decomposed into two Gaussian peaks. The peak at the higher wave number should be the characteristic of the free sulfone stretching mode, while the peak at the lower wave number should be the characteristic of hydrogen-bonded sulfone. The good agreement between the experimental and fitted spectra indicates the reliability of this fitting. A curve-fitting program was employed to quantitatively analyze the spectra regarding the integrated intensity of these separated bands. According to Equation (4), the percentage content of hydrogen bonds in the blend was calculated according to the fitting peak area. The percentage of hydrogen-bonded sulfone group in the PP-3 membrane was 53.04%, and the PP-5 membrane was 62.65%, which is shown in Table 4.

(4) $$F^{H\text{-}bonded}=\frac{A_{H}/a_{R}}{A_{H}/a_{R}+A_{f}}$$

where $A_{f}$ and $A_{H}$ are peak areas corresponding to the free and hydrogen bond methyl groups, respectively, and $a_{R}$ is the free/hydrogen bond CH_3_ unit absorptivity ratio.

### 3.3. Chemical Composition of the Membranes

**Figure 6:**
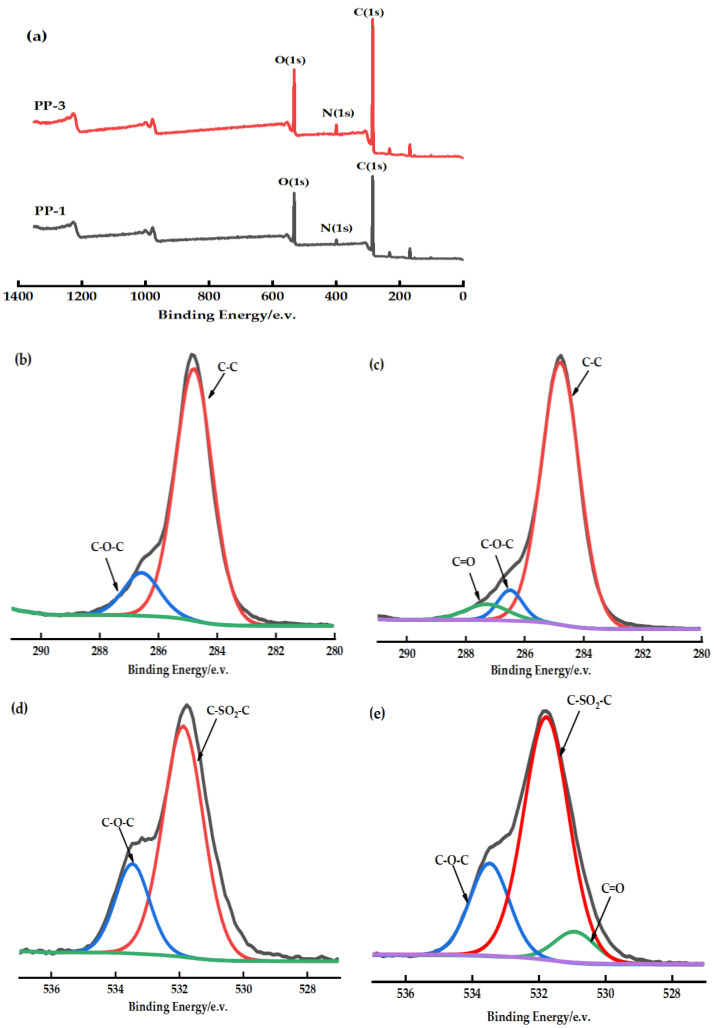
(**a**): XPS full-scan spectra of PP-1 and PP-3 membrane; (**b**,**c**): C1s spectra of PP-1 and PP-3 membrane; (**d**,**e**): O1s spectra of PP-1 and PP-3 membrane.

XPS full-scan spectrum (Figure 6a) shows that the PES/PEI membrane contains oxygen (531 eV), nitrogen (399 eV), and carbon (285 eV). The untreated PES membrane’s C1s high-resolution spectrum was assigned two peaks at BEs of 284.6 eV due to C-C and 286.2 eV due to C-O-C [25] (Figure 6b,c). The deconvolution O1s spectrum of the PES membranes consists of two peaks (Figure 6d,e). Of the two peaks, the main peak at 531.5 eV is the sulfone group (C-SO_2_-C) to which PES belongs, while the second peak observed at 533.2 eV is the C-O-C bond [26]. When the chemical environment of the atoms in the polymer blend changes, new peaks can be observed in the XPS spectrum. A unique and distinctive peak was observed in the high-resolution spectra of PES/PEI membrane C1 and O1 (Figure 6c,e), which was attributed to the carbonyl group (C=O) of PEI. The electron transfer from the proton acceptor to the proton donor causes the electron density in the C–H bond to grow [27]. Therefore, in the XPS spectrum, the binding energy of C1s in the C–H bond is transferred to a lower band. Table 5 lists their atomic compositions. The atomic ratio O/C drops, indicating a decrease in the fraction of oxygen-containing functional groups on the membrane surface. Perhaps the blend increases the flexibility of the polymer molecular chain, and hydrogen bonding causes the bond between the chain segments, thus changing the distribution and concentration of polar groups near the membrane surface.

### 3.4. Crystals of the Membranes

**Figure 7:**
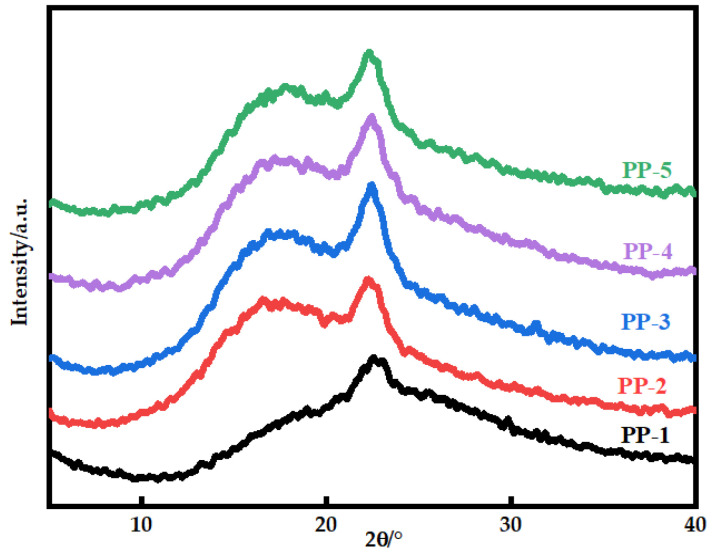
X-ray diffraction patterns of various PES/PEI membranes.

Figure 7 shows the X-ray diffraction patterns of various blended membranes. The PEI polymer chain’s amorphous phase structure results in a broad peak of amorphous material at 18.52° [28]. The addition of PEI makes the crystal diffraction peak intensity of the blended membrane increase at about 22.74°. This could be because the two components can be stacked via π–π interaction and form a hydrogen bond network, causing changes in molecule arrangement [29,30,31]. Decreasing chain entanglement is helpful to promote disorder–order transformation. Table 6 shows the π–π stacking distance of the blended membranes. Because of the shorter π–π stacking distance, molecules can stack more closely, and the interaction between molecules may be strengthened [32].

### 3.5. Membranes Morphology and Microstructure

**Figure 8:**
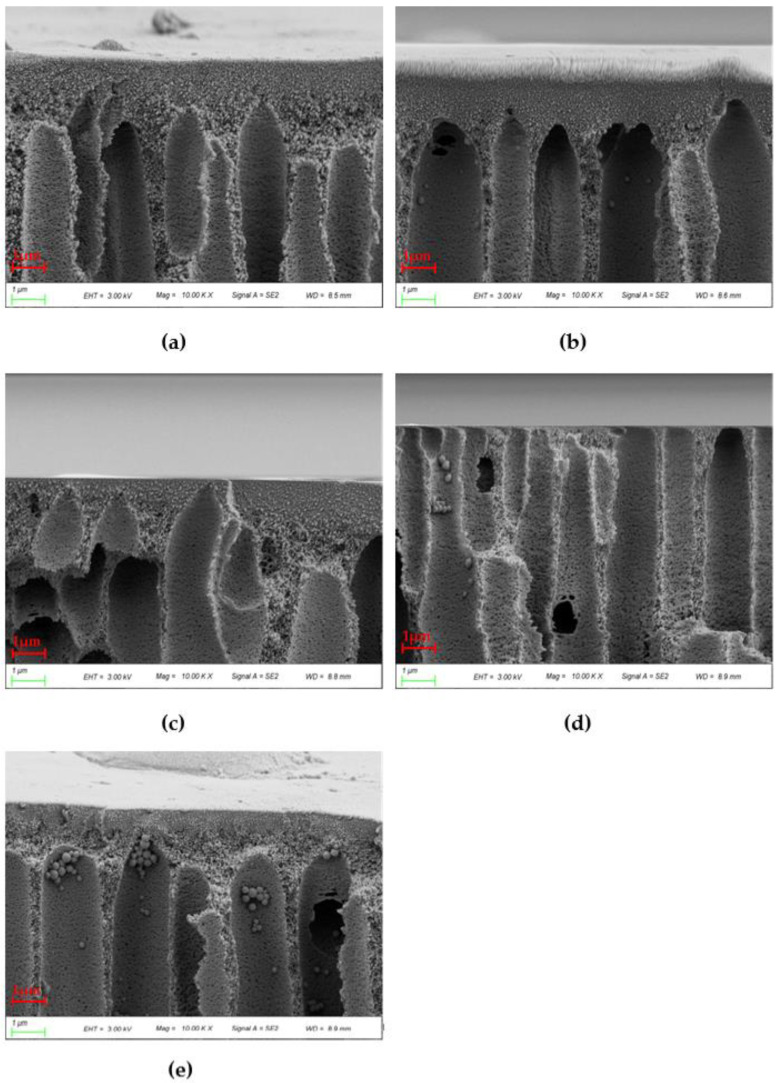
10 × SEM images of cross sections of various PES/PEI membranes: (**a**) PP-1; (**b**) PP-2; (**c**) PP-3; (**d**) PP-4; (**e**) PP-5.

Figure 8 shows the cross sections of various blends. The surface layer of the blend membrane becomes thinner with the increase of PEI content, which is due to the stronger affinity between the coagulating bath and PES/PEI blends compared to PES alone. The exchange rate goes up and the forming conditions are intense because the hydrogen bond between the carbonyl group of water and PEI is stronger than the ether bond between water and PES. When the surface layer forms quickly, the overall exchange time between the coagulating bath and the casting solution climbs, allowing the poor phase of the polymer to develop and coalesce. The phase conversion rate is a variable that determines membrane morphology. The membrane will have a thin surface layer and a porous sublayer with a vast number of macroscopic pores under rapid transformation conditions [33].

Because of the poor compatibility between PES and PEI, the cortex thickened when the PEI content reached 20%. This point was also mentioned in the next chapter’s theoretical computation. The granular structures observed are spherical crystals produced by solid–liquid phase separation. As the mass percent of the blend grows, pre-crystallized aggregates in the casting solution increase.

**Figure 9:**
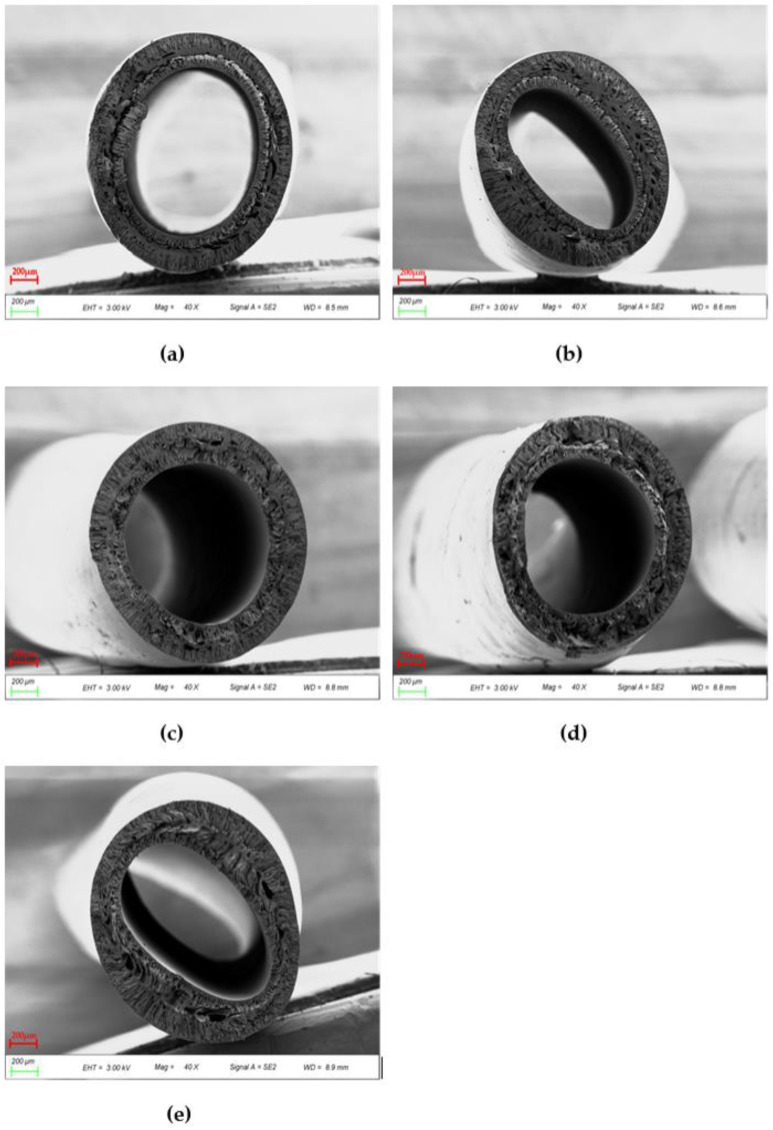
40 × SEM images of cross sections of various PES/PEI membranes: (**a**) PP-1; (**b**) PP-2; (**c**) PP-3; (**d**) PP-4; (**e**) PP-5.

PES pure membrane (PP-1) had sublayers of large finger-like voids extending from the outer and inner surfaces to the middle and regions with spongy structures in the middle of the cross section, as shown in Figure 9. These are related to the slow phase transformation process. Conversely, the membrane eventually transformed into double rows of finger-like pores, with huge pores appearing in the middle of the cross section when PEI was added. The finger-like pores become more numerous as the number of pores in the hollow fiber membrane increases, as does their transmissibility. The appearance of this morphology results from the addition of blends to increase the porosity of the membrane, and the presence of these affects the thermodynamic properties and accelerates the phase separation process [34]. The increase in polymer concentration near the phase conversion interface and the presence of a surface layer induced additional resistance to mass transfer between the coagulation bath and the membrane sublayer, which favors the formation of sublayer pores.

### 3.6. Hydrophilicity of Membranes

**Figure 10:**
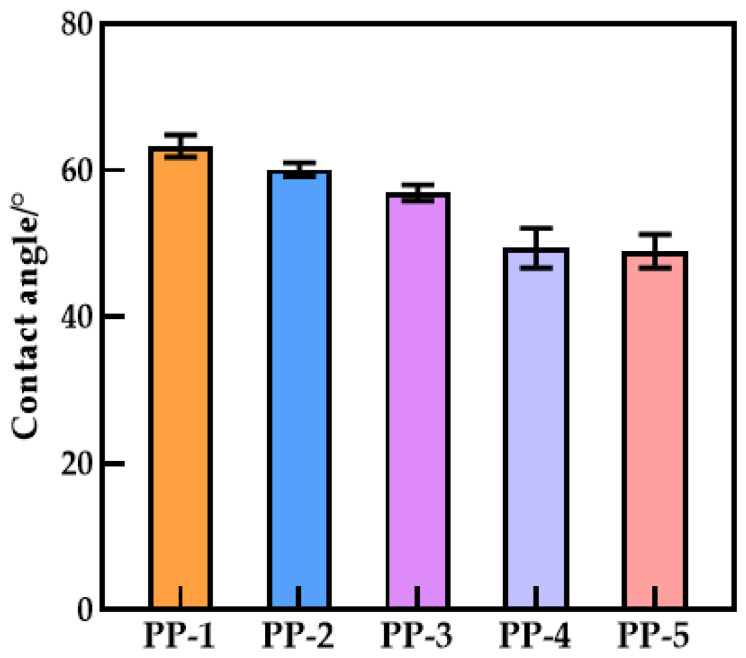
Contact angle of various PES/PEI membranes.

**Figure 11:**
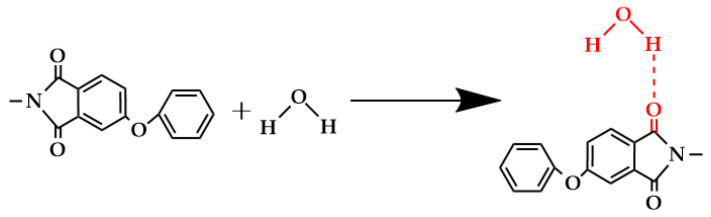
Hydrogen bonding between carbonyl groups and water molecules.

Figure 10 shows that the affinity of the surface of the water-wetted membrane is improved, and the blend improves the hydrophilicity of the hollow fiber membrane. The existence of the carbonyl group and some polar ether groups in PEI will generate a hydrogen bond with a hydrogen atom, resulting in increased hydrophilicity. The shift and broadening of the absorption bands of carbonyl and hydroxyl groups in the infrared spectra were well-confirmed (Figure 4b). The active site (proton acceptor) on the main chain of the polymerization is the imide carbonyl group, while the involvement of ether oxygen is negligible [35,36], as shown in Figure 11. The water contact angle of a 20% PEI blend membrane can reach 48.93°, which is 29.43% lower than a PES membrane. When PEI content reaches 20%, the compatibility with PES becomes worse, so there is little difference between the water contact angle of PP-4 and PP-5 membranes. Overall, the tendency is downward. Wenzel reasoned that the presence of a rough surface would increase the actual solid–liquid contact area above the region shown on the surface geometry, hence, increasing hydrophilicity [37]. According to Equation (5), $\theta_{w}$ is the contact angle of the Wenzel theory; $\theta_{0}$ is Young’s angle of Wenzel’s theory; and r is the ratio of the actual surface area to the apparent surface area of the rough surface. Since r>1, the hydrophilic membrane will become more hydrophilic when the number of pores increases and the roughness increases.

(5) $$\cos\theta_{w}=r\cos\theta_{0}$$

### 3.7. Mechanical Performance of the Membranes

**Figure 12:**
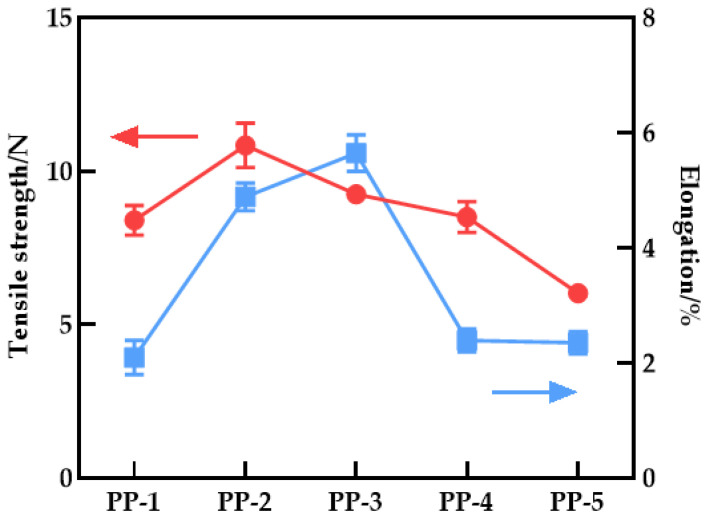
Tensile strength and elongation at break of various PES/PEI membranes.

Figure 12 depicts the effect of PEI content on the maximum strength and elongation at the break of the blend membrane. The maximum strength of the PES/PEI blend membrane increased by 38.34% to 8.84 N, while the elongation at break increased from 2.10% to 5.64%, indicating an overall trend of increase followed by decrease. The overall elongation at break is higher than that of the PES pure membrane. PEI increases the mechanical properties of the blend membrane, possibly due to the hydrogen bond interaction between PES and PEI, forming the blend material hydrogen bond complex. The rigidity of the imide group in the complex can enhance the strength of the blend membrane, while the flexibility of the ether bond can improve the toughness. However, as the PEI content increased, the compatibility declined, and SEM revealed that the porosity increased as well, reducing the mechanical characteristics.

### 3.8. Transport Characteristics of the Membranes

**Figure 13:**
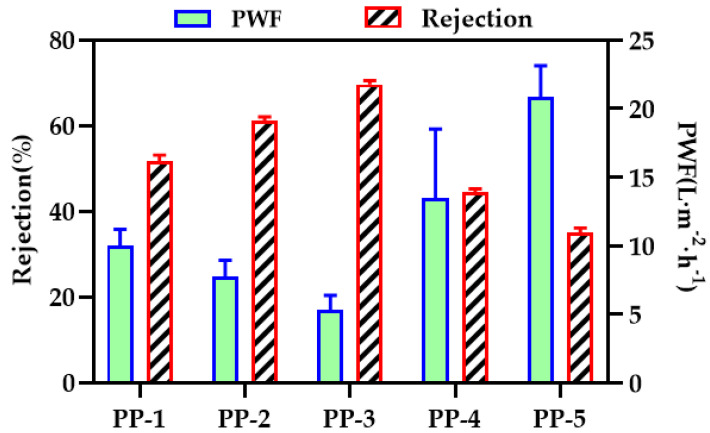
Pure water flux and BSA rejection rates of various PES/PEI membranes.

BSA filtration experiments were performed to evaluate the separation and retention performance of the membranes. The results of permeability flux and BSA rejection are shown in Figure 13. The permeability flux first decreased, then increased, while the trend of the rejection rate was the opposite. A tiny amount of PEI contributes to the growth of PES nucleation, and nucleation is conducive to the formation of a relatively dense film surface structure. On the contrary, when PEI reaches a certain amount, the viscosity of the polymer solution decreases, the phase transformation rate accelerates, and a porous structure with good penetration can occur. Compared with the PES hollow fiber membrane, the rejection rate of the PP-3 blend membrane increased by 18%.

## 4. Compatibility Calculation

Blending compatibility is the ability of multiple substances to beget a uniform and stable system, and it is also the cornerstone of membrane structure regulation. If the Gibbs free energy of the polymer blend casting solution system is less than 0, the two substances are compatible, according to the Flory–Huggins theory. As the entropy of blending of the polymer approaches zero, the Gibbs free energy of the blending system (Equation (6)) is mostly influenced by blending enthalpy [38]. As a result, Schneier et al. deduced the formula for estimating the polymer blend system’s mixing enthalpy (Equation (7)) and determined that the mixing enthalpy’s critical value was 41.86 × 10^−3^ J/mol [39]. Polymer intermolecular forces are classified as dipole $\delta_{p}$, dispersion $\delta_{d}$, and hydrogen bonding $\delta_{h}$. The solubility parameter δ of a polymer can be calculated by the group contribution method (Equation (8)) [40].

(6) ΔG=ΔH−T⋅ΔS<0

(7) $$\Delta H={\left\{X_{1}M_{1}\rho_{1}{\left(\delta_{1}-\delta_{2}\right)}^{2}{\left[\frac{X_{2}}{\left(1-X_{1}\right)M_{1}\rho_{1}+\left(1-X_{2}\right)M_{2}\rho_{2}}\right]}^{2}\right\}}^{1/2}$$

(8) $$\delta ={\left(\delta_{p}{}^{2}+\delta_{d}{}^{2}+\delta_{h}{}^{2}\right)}^{1/2}$$

where ΔG is the Gibbs free energy, kJ/mol; ΔH is the enthalpy of mixing, kJ/mol; T is the absolute temperature, K; ΔS is the entropy of mixing, kJ/ (mol·K); M is the mass fraction, %; M is the molar mass of structural unit, g/mol; ρ is density, g/cm^3^; and δ is the solubility parameter, J^1/2^/mol^3/2^.

**Figure 14:**
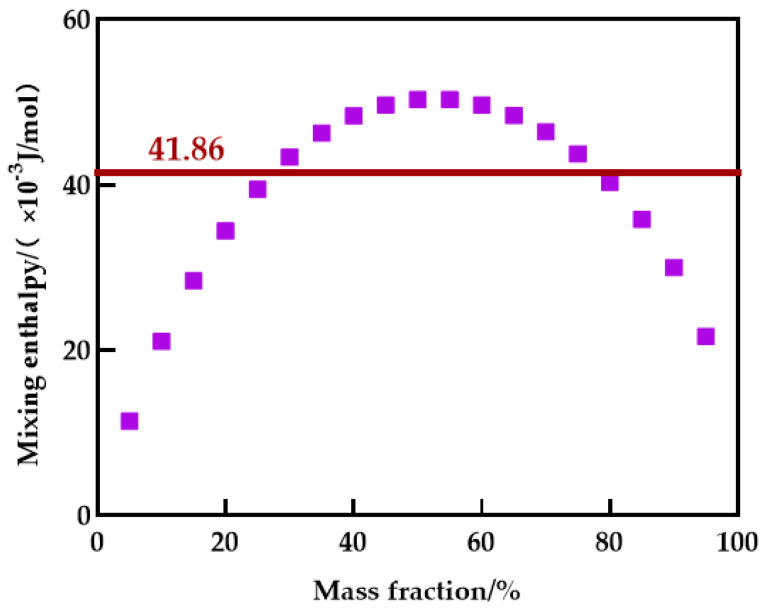
Enthalpy of mixing for blending system.

From Figure 14, the PES/PEI blending system is only compatible within a limited mass fraction range. The blending system can be integrated when the PES/PEI ratio is less than 75/25. In Table 7, the hydrogen bond solubility parameters of the two components are similar, and the hydrogen bond interaction between the two components will promote compatibility. Blending the primary polymer with a polymer capable of forming a hydrogen bond completes the blending membrane.

## 5. Conclusions

Polyethersulfone/polyetherimide hollow fiber blends were successfully prepared by the solution blending method. The compatibility and hydrogen bonding of PES/PEI blends were proved by experimental analysis and theoretical calculation. The blends exhibit single, composition-dependent glass transition temperatures that obey the Fox equation well. The shift of absorption bands of methyl in FTIR spectra provides a basis for use as a hydrogen bond donor. The percentage of hydrogen bonds was calculated by fitting the infrared peaks of the hydrogen bond acceptor sulfone group. The structure exhibits a C−H···O interaction motif. According to the Flory–Huggins theory, the blending system can be integrated when the PES/PEI ratio is less than 75/25. The strong hydrogen bond between the carbonyl group of polyetherimide and water accelerated the phase separation process, and the blended membrane gradually changed to a double-row finger-hole shape. The hydrophilicity of the membrane was also effectively enhanced. It can be found that the PP-3 blend membrane has the best overall performance, with a maximum force of 7.25 N, an elongation at a break of 5.24%, and an interception capacity of 69.66%.

## Figures and Tables

**Table 1:** Chemical structure of the polymer.

Polymer	Chemical Structure
Polyethersulfone	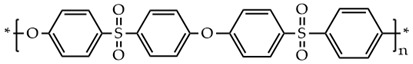
Polyetherimide	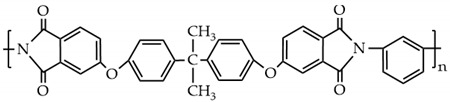

**Table 2:** The Molecular weight of polymers.

Polymer	M_W_	M_N_	M_W_/M_N_
Polyethersulfone	75,000	39,000	1.9
Polyetherimide	50,000	25,000	2

**Table 3:** The specific ratio of casting solution.

Code	PES/PEI (27 wt.%)	DMAc (68 wt.%)	PVP (5 wt.%)
PP-1	100/0	68	5
PP-2	95/5	68	5
PP-3	90/10	68	5
PP-4	85/15	68	5
PP-5	80/20	68	5

**Table 4:** Data from FTIR fitting integral spectra of PP-3 and PP-5 membranes.

Sample	Total Area of -SO_2_^−^	Area of H-Bonded -SO_2_^−^	Area of Free -SO_2_^−^	*F ^H-bonded^* (%)
PP-3	2239.14	1187.75	1051.39	53.04
PP-5	4274.88	2678.30	1596.58	62.65

**Table 5:** Elemental composition by atomic percent of different kinds of PES/PEI membranes.

Membrane	C (%)	O (%)
PP-1	75.06	20.79
PP-3	77.76	17.59

**Table 6:** π–π stacking distance of various blend membranes.

Membrane	2 θ/°	d _(π–π)_/Å
PP-2	22.02	4.03
PP-3	22.38	3.97
PP-4	22.48	3.95
PP-5	22.62	3.93

**Table 7:** Main parameters of blend polymers.

Polymer	$\delta_{p}$	$\delta_{d}$	$\delta_{h}$	δ	ρ	M
PEI	3.64	10.33	3.45	11.48	1.27	622
PES	5.08	8.60	3.81	10.70	1.44	232

## Data Availability

The data used to support the findings of this study are available from the corresponding author upon request.
